# Hierarchical integration of mNGS, PCR, and other conventional methods for precision TB diagnostics

**DOI:** 10.1128/spectrum.01931-25

**Published:** 2025-09-11

**Authors:** Yating Zhao, Longting Du, Junli Song, Wei Sun, Yili Chen, Xuegao Yu, Hao Huang, Gang Huang, Enpu Huang, Ni Wang, Shu An, Lu Ai, Peisong Chen

**Affiliations:** 1 Department of Laboratory Medicine, The First Afﬁliated Hospital of Sun Yat-sen University, Guangzhou, Guangdong, China; 2 Reproductive Medicine Center, The First Afﬁliated Hospital of Sun Yat-sen University, Guangzhou, Guangdong, China; 3 Department of Laboratory Medicine, Maternal and Child Health Hospital of Qingyuan City, Qingyuan, Guangdong, China; 4 Organ Transplant Center, The First Affiliated Hospital of Sun Yat-sen University, Guangzhou, Guangdong, China; 5 Department of Laboratory Medicine, The First People’s Hospital of Fangchenggang City, Fangchenggang, Guangxi, China; 6 Department of Laboratory Medicine, The Second People’s Hospital of Qujing City, Qujing, Yunnan, China; Children's National Hospital, George Washington University, Washington, DC, USA

**Keywords:** mNGS, ddPCR, RT-qPCR, EasyNAT, MTBC

## Abstract

This study systematically compared the diagnostic accuracy of seven assays for detecting the *Mycobacterium tuberculosis* complex, including metagenomic next-generation sequencing (mNGS), droplet digital polymerase chain reaction, real-time quantitative polymerase chain reaction, EasyNAT MTC, GeneXpert MTB/RIF, interferon-gamma release assay (IGRA), and acid‒fast staining (AFS). We try to select appropriate combinations of tuberculosis (TB) detection methods for regions with varying levels of medical resources, based on sensitivity, cost-effectiveness, and operational feasibility. A retrospective analysis was conducted on 141 samples collected from patients with suspected active TB at The First Affiliated Hospital of Sun Yat-sen University between April 2022 and April 2024. Among these samples, there were 100 cases assigned to the case group and 41 cases to the control group, based on the tuberculosis diagnostic criteria. Historical data for Xpert, IGRA, and AFS were collected, and parallel experiments using mNGS, droplet digital PCR (ddPCR), real-time quantitative polymerase chain reaction (RT-qPCR), and EasyNAT were conducted on all samples. Diagnostic performance was evaluated by comparing it with the final clinical diagnoses. Sensitivity, specificity, positive predictive value, negative predictive value, and receiver operating characteristic (ROC) curve analysis were conducted, along with DeLong tests for statistical comparison. Compared with the final clinical diagnosis, mNGS demonstrated the highest sensitivity (100%), followed by IGRA (79.2%), EasyNAT (79.1%), RT-qPCR (78.0%), ddPCR (75.8%), Xpert (75.3%), and AFS (16.7%). The specificity was 100% for both Xpert and AFS, followed by ddPCR (97.6%), RT-qPCR (95.1%), EasyNAT (92.7%), IGRA (72.7%), and mNGS (75.6%). ROC analysis revealed a significantly greater area under the ROC curve for mNGS (0.878) than for ddPCR (0.817, *P* = 0.031). DeLong tests revealed statistically significant differences in diagnostic performance between mNGS and ddPCR (*P* < 0.05) and between IGRA and AFS (*P* < 0.01). mNGS uniquely identified the pathogens involved in co-infection and quantified pathogen-specific sequencing reads. Through a comprehensive evaluation of the diagnostic efficacy, cost-effectiveness, and timeliness of tuberculosis detection methods, we propose corresponding combinations of TB testing approaches for regions with different healthcare resources. For undeveloped regions with limited resources, a combination of AFS +EasyNAT + chest X-ray is recommended. Primary care facilities may additionally employ IGRA + RT-qPCR. Intermediate-level hospitals can incorporate Xpert MTB/RIF for drug resistance testing, while tertiary hospitals or specialized centers should, on the basis of these fundamental tests, utilize mNGS for diagnosis and ddPCR for therapeutic monitoring in patients with complex mixed infections.

**IMPORTANCE:**

This study is the first to comprehensively evaluate the diagnostic efficacy, cost-effectiveness, and timeliness of seven TB detection methods in a single-center cohort. Our findings provide actionable solutions for optimizing TB diagnostics in diverse healthcare ecosystems, aligning with the WHO’s End TB Strategy to ensure equitable access to rapid diagnostics.

## INTRODUCTION

Tuberculosis (TB), caused by the *Mycobacterium tuberculosis* complex (MTBC), remains a major global health challenge, with an estimated 10.8 million (95% uncertainty interval: 10.1–11.7 million) incident cases globally in 2024, disproportionately affecting low- and middle-income countries (1, 2). Pulmonary tuberculosis (PTB) constitutes approximately 90% of active cases (3). PTB progression is insidious and clinically non-specific, contributing to delays in accurate diagnosis and timely intervention (4–6). China, which ranks third in terms of the global TB burden, faces compounded diagnostic hurdles in impoverished regions where healthcare resources are scarce and socioeconomic disparities exacerbate disease transmission (3, 7).

Traditional laboratory methods, including acid‒fast staining (AFS) and mycobacterial culture, remain foundational but are essential in rural and resource-limited settings owing to high costs and infrastructural demands (8). AFS has poor sensitivity (15%–40%) and high rates of false negatives, whereas culture-based identification requires 2–6 weeks for colony growth, rendering it impractical for acute clinical decision-making (9–11).

Interferon-gamma release assays (IGRAs), which detect MTBC-specific immune responses, lack diagnostic accuracy in distinguishing latent infection from active disease (12). These shortcomings underscore the urgent need for rapid, precise diagnostic alternatives.

Nucleic acid amplification tests have revolutionized TB diagnostics (8, 13). Real-time quantitative polymerase chain reaction (RT-qPCR) combines TaqMan fluorescent probe quantitative technology to detect TB, providing high specificity, repeatability, and simple operation. The Xpert MTB/RIF (Xpert) assay (Cepheid, Sunnyvale, CA, USA), endorsed by the World Health Organization (WHO) for first-line testing, enables simultaneous MTBC detection and rifampicin resistance screening within 2 h. However, its sensitivity decreases to 67% in smear-negative specimens (14, 15). The EasyNAT MTC assay (USTAR Biotechnologies, Hangzhou, China), a loop-mediated isothermal amplification-based tuberculosis molecular assay, has been recommended for diagnosing active pulmonary tuberculosis in primary care settings and added to the first WHO List of Essential In Vitro Diagnostics (8, 16). It provides comparable sensitivity at a lower cost and with a processing time of less than 2 h, making it suitable for resource-limited settings (17).

Metagenomic next-generation sequencing (mNGS) provides culture-independent, unbiased pathogen detection, enabling early identification of MTBC and pathogens involved in co-infection (13, 18–20). Typically, when the number of MTBC reads meets or even exceeds the reporting threshold, as little as one read, it is considered a positive report (21, 22). Despite its diagnostic promise, mNGS faces several challenges. Firstly, it involves prolonged turnaround times, typically taking 24–48 h, along with bioinformatics complexity and difficulty in distinguishing true positives from contamination or analytical artifacts. Moreover, the most important limitation of mNGS in diagnosis is that it is still not a first-line diagnostic assay, and the detection of irrelevant organisms may cause confusion for physicians (23, 24).

Recently, droplet digital PCR (ddPCR) has been developed and used for PTB because of its unparalleled sensitivity and precision (25). Even for trace DNA in samples at low concentrations, ddPCR still has high accuracy (26, 27). Taking the Targeting One Droplet Digital PCR System as an example, primer pairs were designed to detect the conserved regions of the insert sequences (IS) 6110 and 1081 of MTBC. A single copy in any droplet qualifies as positive, achieving near-absolute specificity (21). While promising, ddPCR lacks comprehensive performance data compared with established assays (25).

In this study, we performed a single-center study involving patients with suspected PTB infections evaluating seven diagnostic methods—AFS, IGRA, Xpert, EasyNAT, mNGS, RT-qPCR, and ddPCR. We try to select appropriate combinations of tuberculosis detection methods for regions with varying levels of medical resources, based on sensitivity, cost-effectiveness, and operational feasibility.

## MATERIALS AND METHODS

### Study design and participants

**Fig 1:**
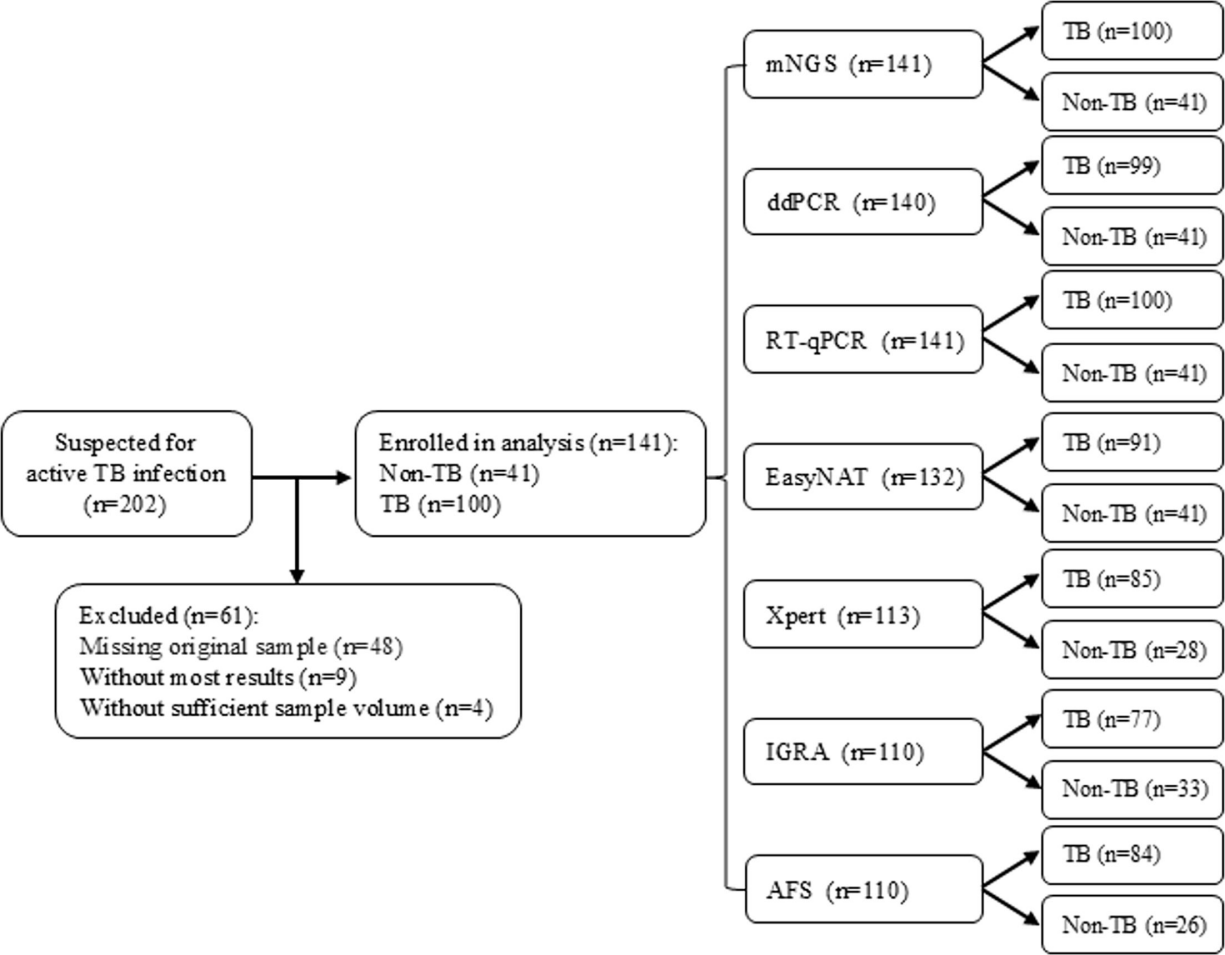
Study design.

A total of 141 samples from 139 patients suspected of having TB infection were enrolled at The First Affiliated Hospital of Sun Yat-sen University between March 2022 and April 2024. The inclusion criteria were as follows: (i) clinical symptoms persisting ≥2 weeks (e.g., productive cough, prolonged fever, night sweats, and unexplained weight loss); (ii) radiographic abnormalities consistent with TB (e.g., cavitary lesions and nodular infiltrates); and (iii) age ≥5 years to ensure adequate sputum and/or bronchoalveolar lavage fluid (BALF) specimen collection. The specimens were either processed immediately or stored at −80°C for subsequent analyses. Sixty-one patients in the initial cohort were excluded because of incomplete diagnostic data (e.g., missing assay results), non-compliant samples, or sample degradation (Fig. 1). Diagnostic results for AFS, IGRA, and Xpert were retrieved retrospectively from the laboratory information system. mNGS, EasyNAT MTC, RT-qPCR, and ddPCR were prospectively performed on the available specimens. Final TB diagnoses were established by a multidisciplinary panel of infectious disease and respiratory specialists, adhering to the following criteria: (i) chest imaging showing lesions consistent with active MTBC accompanied by (a) suspicious symptoms, (b) moderate or above positive tuberculin test, (c) positive IGRA test, (d) positive tuberculosis antibody, (e) pathological changes in extrapulmonary tissues, and (f) bronchoscopy consistent with active MTBC; (ii) double sputum smears with positive AFS one sputum smear with positive AFS accompanied by chest imaging evidence or culture-confirmed MTBC; (iii) culture-confirmed MTBC with chest imaging evidence; (iv) molecular biology confirmation with chest imaging evidence; and (v) pathological changes in pulmonary tissue. Demographic data (age and sex) and specimen types were systematically recorded for all included patients.

### Sample processing

For non-viscous BALF specimens, they were gently homogenized, and 1 mL was directly transferred to a 1.5 mL microcentrifuge tube. However, for mucus-rich BALF specimens or sputum, digestive solution was added to a 1.5 mL centrifuge tube containing 0.2–0.3 mL specimen, which were mechanically disrupted via 20 aspiration cycles vortexed vigorously (≥10 vertical shaking cycles) and then incubated at 37°C in a thermal block for 3 min with intermittent agitation. Afterward, the specimens were centrifuged briefly, and 1 mL of completely liquefied specimens was transferred into a fresh 1.5 mL centrifuge tube. For venous blood, the specimens were centrifuged at 1,600 × *g* for 10 min, and 0.3 mL plasma fraction was aspirated from the supernatant. For cerebrospinal fluid (CSF) or tracheal secretions with preservation solution, gentle vortex mixing was performed, followed by precise transfer of 0.6 mL to a 1.5 mL microcentrifuge tube. For other specimens such as pleural effusion, drainage or perfusion fluid, and peritoneal dialysis fluid, the specimens were centrifuged at 1,600 × *g* for 10 min; a large amount of supernatant was carefully discarded; and the remaining 5 mL of supernatant was used to resuspend the precipitated cells. Then, 1 mL was pipetted into a 1.5 mL centrifuge tube and centrifuged at 12,000 × *g* for 5 min; 0.9 mL of the supernatant was discarded; and 0.1 mL of precipitate was left for later use.

### mNGS

All the samples required a host depletion approach prior to nucleic acid extraction. Following the manufacturer’s operational manual, DNA was extracted via the IDseq Micro DNA Kit (Visual Medicine, Guangzhou, China). Except for EasyNAT, which requires the direct utilization of processed raw samples, the DNA extraction for all other methods follows the extraction procedure of mNGS. A DNA library was constructed via a transposase-based method. After purification and size selection, a Qubit instrument was used to detect the concentration of the library before loading. The library was subsequently sequenced on the Illumina Next Seq 550 system via a 75 bp single-end sequencing kit (Illumina, California, USA). The qualified data were that no fewer than 10 million reads were obtained for each sample, and the Q30 score was ≥85%. During each sequencing, the negative control was subjected to parallel processing and sequencing for quality control (28). The detection of one specific *Mycobacterium tuberculosis* sequence can be used to determine positivity (29).

### RT-qPCR

*Mycobacterium tuberculosis* complex nucleic acid detection kits (PCR-fluorescent probes) (Daan Gene, Guangzhou, China) provided the reagents needed for RT-qPCR. By isothermal amplification on an Applied Biosystems 7500 Fast Real-Time PCR System (USA), we obtained a cycle threshold (CT) for each sample and defined positive reports as CT values of ≤38. Notably, both negative and positive controls were required for the tests.

### EasyNAT

*Mycobacterium tuberculosis* complex nucleic acid detection kits (isothermal amplification-real-time fluorescence) (USTAR Biotechnologies) were used to detect the IS 6110 region of the genomic DNA. The extraction solution was mixed thoroughly until there was no sediment and was transferred to the internal standard tube. Then it was mixed thoroughly after 10–20 s. The above mixture was mixed with the sample and transferred to the detection tube. The tube cap was tightened, and the tube was placed on the automatic integrated nucleic acid amplification detection analyzer (USTAR Biotechnologies). The analyzer can automatically report positive results (MTC Tt ≤40) or negative results (MTC Tt >40). The samples needed to be retested if the analyzer showed invalid results because of inappropriate operation or other reasons.

### ddPCR

ddPCR was performed via the Targeting One Droplet Digital PCR System as previously described (21). A 30 µL reaction mixture and 180 µL of oil were passed through the Targeting One Drop Maker M1 (Targeting One, Beijing, China) to generate emulsion droplets. After PCR amplification via a thermal cycler, the droplets were then loaded onto the Chip Reader R1 (Targeting One) and scanned for analysis via Targeting One analysis software (Targeting One) for each droplet. Finally, the FAM (488 nm laser) fluorescence intensity of IS1081 and the VIC (532 nm laser) fluorescence intensity of IS6110 were analyzed for each sample. Positive and negative controls were used to determine the threshold level of fluorescence for selecting positive droplets. MTBC infection was confirmed when at least three copies per reaction of the IS6110 or IS1081 gene were positive (30).

### Statistical analysis

SPSS 26.0 software was used for statistical analysis. Continuous data with a normal distribution were described as the mean ± standard deviation, whereas continuous data with non-normal distribution were described as the median (interquartile range). Categorical variables were represented by case numbers (*n*) and percentages (%) and were analyzed by the chi-square test. Receiver operating characteristic (ROC) analysis and DeLong tests were used to evaluate the diagnostic efficacy of different methods.

## RESULTS

### Baseline characteristics of the participants

A total of 141 samples were enrolled from 139 patients suspected of having TB infection. Overall, 100 samples were obtained from patients with a confirmed clinical diagnosis. The baseline characteristics of patients are presented in Table 1. The average age of the TB-infected patients was 56.7 years, and 68 (68%) were men. In the non-TB group, the average age was 56.6 years, and 31 (75.6%) patients were men. There was no statistically significant difference in age or sex. The types of samples were mostly BALF (81, 81%), followed by venous blood (6, 6.0%), CSF (1, 1%), sputum (1, 1%), tracheal secretions (2, 2%) and others (9, 9%) in the TB group. There were all BALF samples in the non-TB group.

**TABLE 1:** Baseline characteristics

Features	TB (*n* = 100)	Non-TB (*n* = 41)
Age (years, mean ± SD)	56.7 ± 18.1	56.6 ± 23.0
Gender (male), *n* (%)	68 (68.0)	31 (75.6)
Types of samples, *n* (%)		
Bronchoalveolar lavage fluid	81 (81.0)	41 (100)
Venous blood	6 (6.0)	0
Cerebrospinal fluid	1 (1.0)	0
Sputum	1 (1.0)	0
Tracheal secretions	2 (2.0)	0
Others (pleural effusion, drainage or perfusion fluid, tissue, pus, and peritoneal dialysis fluid)	9 (9.0)	0

### Performance of mNGS, ddPCR, RT-qPCR, EasyNAT, Xpert, IGRA, and AFS for TB detection

Compared with the final clinical diagnosis determined by physicians, we evaluated the performance of mNGS, ddPCR, RT-qPCR, EasyNAT, Xpert, IGRA, and AFS for TB detection and assessed the sensitivity and specificity of each test. As shown in Table 2, the sensitivities of mNGS, ddPCR, RT-qPCR, EasyNAT, Xpert, IGRA, and AFS for TB detection were 100.0%, 75.8%, 78.0%, 79.1%, 75.3%, 79.2%, and 16.7%, respectively, and the specificities were 75.6%, 97.6%, 95.1%, 92.7%, 100.0%, 72.7%, and 100.0% respectively. The positive predictive values were 90.9%, 98.7%, 97.5%, 96.0%, 100.0%, 87.1%, and 100.0%, respectively, while the negative predictive values (NPVs) were 100.0%, 62.5%, 63.9%, 66.7%, 57.1%, 60.0%, and 27.1%, respectively. mNGS demonstrated a specificity of 75.6% (31 out of 41), with 10 false-positive cases identified.

**TABLE 2:** Performance of mNGS, ddPCR, RT-qPCR, EasyNAT, Xpert, IGRA, and AFS for TB detection compared with the final clinical diagnosis^*a*^

Methods		Available sample (*n*)	Final diagnosis		Sensitivity (%) (95% CI)	Specificity (%) (95% CI)	PPV (%) (95% CI)	NPV (%) (95% CI)
			+	−				
mNGS	+	141	100	10	100 (100/100) (100–100)	75.6 (31/41) (68.9–82.3)	90.9 (100/110) (85.5–96.3)	100.0 (31/31) (100–100)
	−		0	31				
ddPCR	+	140	75	1	75.8 (75/99) (67.4–84.2)	97.6 (40/41) (92.9–100.0)	98.7 (75/76) (96.2–100.0)	62.5 (40/64) (50.6–74.4)
	−		24	40				
RT-qPCR	+	141	78	2	78.0 (78/100) (69.9–86.1)	95.1 (39/41) (88.5–100.0)	97.5 (78/80) (94.1–100.0)	63.9 (39/61) (51.8–76.0)
	−		22	39				
EasyNAT	+	132	72	3	79.1 (72/91) (70.7–87.5)	92.7 (38/41) (84.8–100.0)	96.0 (72/75) (91.6–100.0)	66.7 (38/57) (54.5–78.9)
	−		19	38				
Xpert	+	113	64	0	75.3 (64/85) (66.1–84.5)	100.0 (28/28) (100–100)	100.0 (64/64) (100–100)	57.1 (28/49) (43.2–71.0)
	−		21	28				
IGRA	+	110	61	9	79.2 (61/77) (70.1–88.3)	72.7 (24/33) (57.5–87.9)	87.1 (61/70) (79.2–95.0)	60.0 (24/40) (44.8–75.2)
	−		16	24				
AFS	+	110	14	0	16.7 (14/84) (8.7–24.7)	100.0 (26/26) (100–100)	100.0 (14/14) (100–100)	27.1 (26/96) (18.2–36.0)
	−		70	26				

^*a*^ CI, confidence interval; NPV, negative predictive value; PPV, positive predictive value.“+” means positive, and “−” means negative.

**Fig 2:**
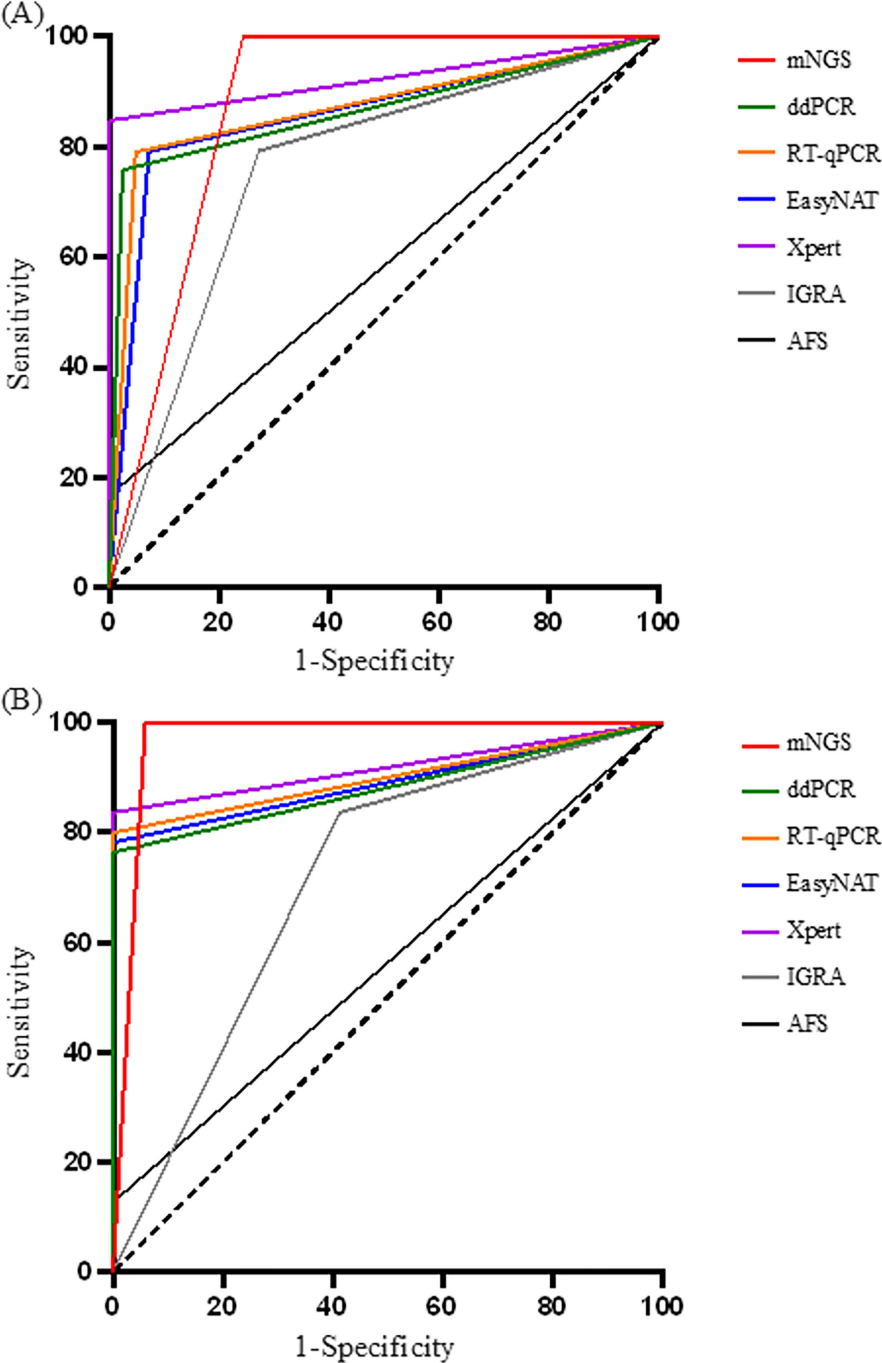
Receiver operating characteristic (ROC) curve of mNGS, ddPCR, RT-qPCR, EasyNAT, Xpert, IGRA, and AFS on TB in all samples (*n* = 141, 100 positive and 41 negative samples) (**A**) and in samples with complete data (*n* = 72, 55 positive and 17 negative samples) (**B**).

The ROC curve was also generated and is shown in Fig. 2 and Table 3. The areas under the receiver operating characteristic curve (AUCs) were 0.878, 0.867, 0.871, 0.859, 0.924, 0.760, and 0.583, respectively (Fig. 2A). When complete data were retained (*n* = 72; 55 positive and 17 negative samples), the AUCs (shown as AUC*) were 0.971, 0.882, 0.900, 0.891, 0.918, 0.712, and 0.564 (Fig. 2B). The DeLong test was used to determine the significance of the AUCs (Table 4). No doubt, mNGS showed the best diagnostic accuracy. There were differences between mNGS and ddPCR. The differences among ddPCR, RT-qPCR, EasyNAT, and Xpert were not statistically significant.

**TABLE 3:** AUCs of mNGS, ddPCR, RT-qPCR, EasyNAT, Xpert, IGRA, and AFS for TB detection^*b*^

Methods	AUC (95% CI)	AUC^*a*^ (95% CI)
mNGS	0.878 (0.798–0.958)	0.971 (0.905–1.000)
ddPCR	0.867 (0.806–0.927)	0.882 (0.806–0.958)
RT-qPCR	0.871 (0.808–0.933)	0.900 (0.831–0.970)
EasyNAT	0.859 (0.791–0.928)	0.891 (0.818–0.964)
Xpert	0.924 (0.875–0.972)	0.918 (0.855–0.981)
IGRA	0.760 (0.657–0.863)	0.712 (0.560–0.865)
AFS	0.583 (0.467–0.699)	0.564 (0.416–0.711)

^*a*^ In samples with complete data (*n* = 72; 55 positive and 17 negative samples).

^*b*^ AUC, area under the receiver operating characteristic curve.

**TABLE 4:** DeLong test results of the AUCs

	*Z*	*P*	Δ AUC	95% CI	
				Lower limit	Upper limit
mNGS–ddPCR	2.153	0.031^*a*^	0.089	0.008	0.170
mNGS–RT-qPCR	1.762	0.078	0.071	−0.008	0.149
mNGS–EasyNAT	1.959	0.050	0.080	0.000	0.159
mNGS–Xpert	1.354	0.176	0.052	−0.023	0.128
mNGS–IGRA	3.323	0.001^*b*^	0.258	0.106	0.411
mNGS–AFS	10.958	0.000^*c*^	0.407	0.334	0.480
ddPCR–RT-qPCR	−0.629	0.529	−0.018	−0.075	0.038
ddPCR–EasyNAT	−0.331	0.741	−0.009	−0.063	0.045
ddPCR–Xpert	−1.070	0.284	−0.036	−0.103	0.030
ddPCR–IGRA	2.263	0.024^*a*^	0.170	0.023	0.316
ddPCR–AFS	9.037	0.000^*c*^	0.318	0.249	0.387
RT-qPCR–EasyNAT	0.299	0.765	0.009	−0.050	0.069
RT-qPCR–Xpert	−0.497	0.619	−0.018	−0.090	0.054
RT-qPCR–IGRA	2.540	0.011^*a*^	0.188	0.043	0.333
RT-qPCR–AFS	9.760	0.000^*c*^	0.336	0.269	0.404
EasyNAT–Xpert	−0.830	0.407	−0.027	−0.092	0.037
EasyNAT–IGRA	2.400	0.016^*a*^	0.179	0.033	0.324
EasyNAT–AFS	9.388	0.000^*c*^	0.327	0.259	0.396
Xpert–IGRA	2.874	0.004^*b*^	0.206	0.065	0.346
Xpert–AFS	11.473	0.000^*c*^	0.355	0.294	0.415
IGRA–AFS	2.053	0.040^*a*^	0.149	0.007	0.291

^*a*^ *P* < 0.05.

^*b*^ *P* < 0.01.

^*c*^ *P* < 0.001.

### Identification of the pathogens involved in co-infection in mNGS

**Fig 3:**
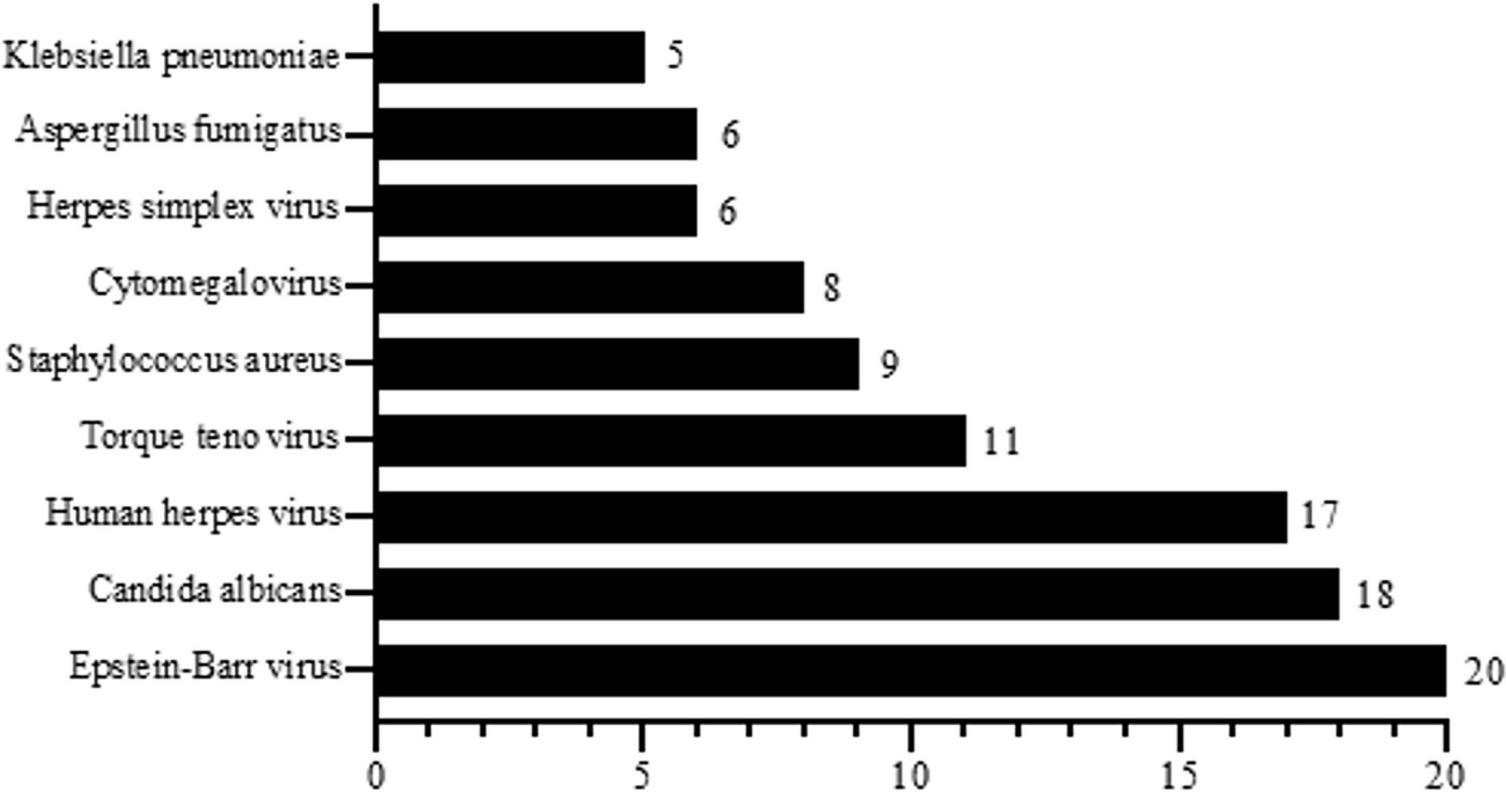
Co-infection pathogens detected by mNGS in TB patients.

**Fig 4:**
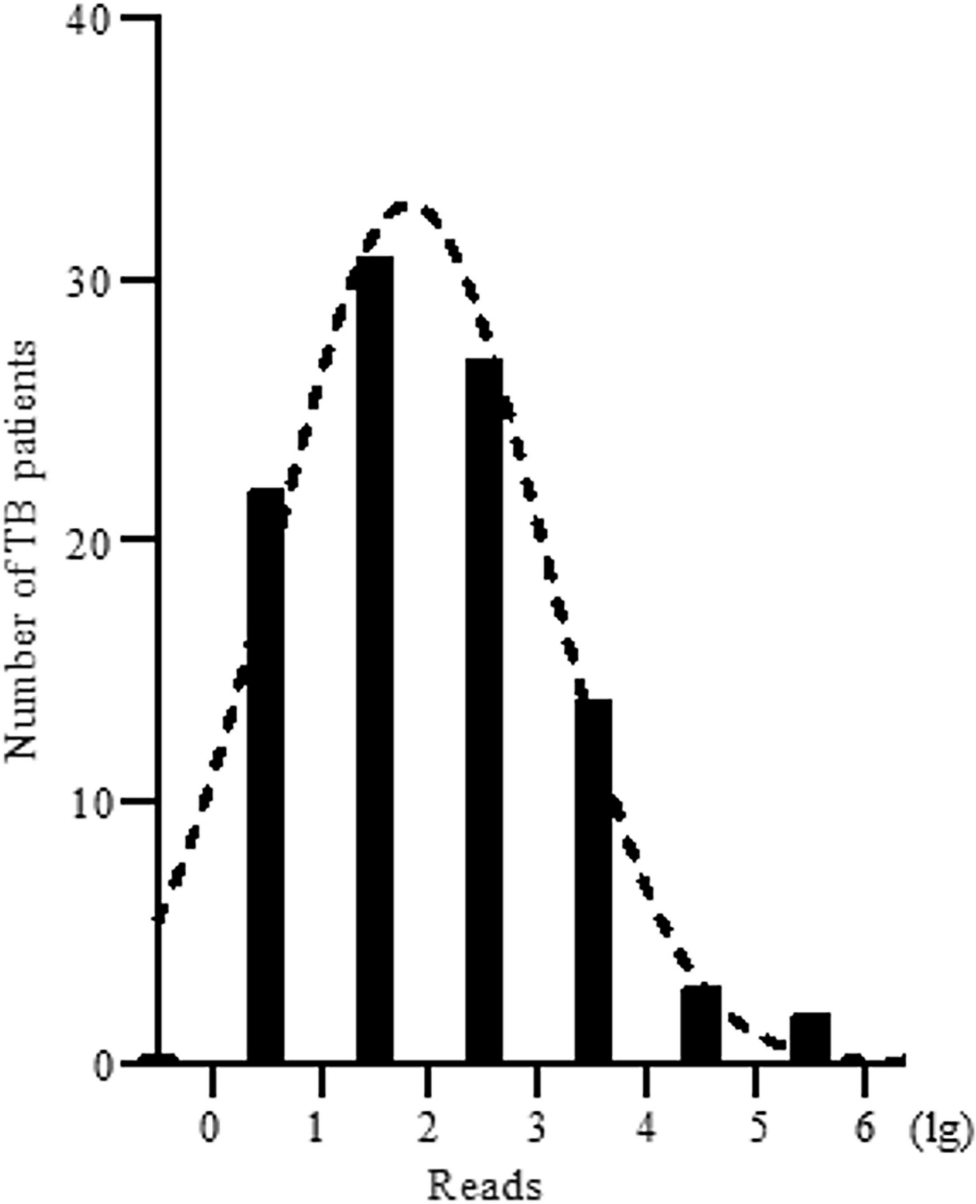
Frequency distribution of MTBC reads detected by metagenomic next-generation sequencing in tuberculosis (TB) patients.

The principal advantage of mNGS lies in its capacity to detect the pathogens involved in co-infection comprehensively within a single assay while providing quantitative sequence read data (Fig. 3 and 4). Our analysis identified nine prevalent pathogens in TB patients, as illustrated in Fig. 3. The prevalence of viral co-infections is high, predominantly involving Epstein-Barr virus (20 out of 100), human herpes virus (17 out of 100), and Torque teno virus (11 out of 100). These viruses have high prevalence rates and also exist in healthy individuals. Fungal pathogens were predominantly *Candida albicans* (18 out of 100) and *Aspergillus fumigatus* (6 out of 100), which are common in immunocompromised patients. Additionally, bacterial co-infections included gram-positive cocci such as *Staphylococcus aureus* (9 out of 100) and gram-negative bacilli such as *Klebsiella pneumoniae* (5 out of 100), both of which were frequently associated with hospital-acquired infections. A pronounced right-skewed distribution is observed for MTBC sequences, with a majority of specimens (frequency peak) clustering in the higher read-count intervals (Fig. 4). The lower bound of detection (reads) demonstrates the ability of mNGS to identify low-abundance taxa, although the clinical relevance of such findings requires further validation.

### Time and cost of each method in local hospitals

We compiled the costs and time for reporting the results of mNGS, ddPCR, RT-qPCR, EasyNAT, Xpert, IGRA, and AFS for TB detection, as shown in Table 5. The reporting times of EasyNAT, Xpert, and AFS were within 2 h, which was faster than those of RT-qPCR (4 h), IGRA (24 h), mNGS, and ddPCR (24–48 h) because of simple specimen pretreatment and easy operation. AFS has the lowest cost, followed by RT-qPCR and EasyNAT, ddPCR, IGRA, Xpert, and mNGS. Because ddPCR has not yet been put into clinical use on a large scale, we only gave a reference price.

**TABLE 5:** Time and cost of mNGS, ddPCR, RT-qPCR, EasyNAT, Xpert, IGRA, and AFS for TB detection

Methods	Time (h)	Cost (¥)
mNGS	24–48	2,870.4
ddPCR	24–48	200.0
RT-qPCR	Within 4	73.6
EasyNAT	Within 2	73.6
Xpert	Within 2	783.0
IGRA	Within 24	400.0
AFS	Within 2	15.8

## DISCUSSION

TB continues to be the foremost cause of mortality globally from a single infectious agent, with approximately a quarter of the world’s population (approximately 2 billion individuals) exhibiting an immune response to MTBC in the absence of clinical, microbiological, or radiological evidence, a condition termed latent tuberculosis infection (LTBI) that entails a lifelong risk of reactivation, leading to TB disease in 5%–10% of cases. The WHO advocates IGRA for LTBI diagnosis because of its high specificity in bacillus Calmette–Guérin-vaccinated populations (12, 31). However, our study revealed suboptimal IGRA performance (sensitivity: 79.2%, specificity: 72.7%, and NPV: 60%), particularly in perinatal TB cases, underscoring the necessity for complementary diagnostic methods such as chest X-ray and molecular assays in clinical workflows (31–33).

Despite its low cost and operational simplicity, AFS has poor sensitivity (specificity: 100%), rendering it inadequate as a stand-alone test. When patients have a history of tuberculosis infection, non-tuberculous mycobacterial infection, or *Nocardia* infection, AFS may produce false-positive results. A 2021 nationwide survey conducted in 17 designated tuberculosis hospitals in China, which focused on acid–fast bacillus smear-positive patients, revealed that the proportion of non-tuberculous mycobacteria (NTMs) was 6.8% (34). Moreover, the high-risk populations for NTM infection primarily included solid organ transplant recipients and allogeneic hematopoietic stem cell transplant recipients (35). However, owing to the low incidence of NTM and randomization factors, NTM cases were not included in this study, which contributed to a relatively high specificity.

Culture-based methods, although definitive, suffer from prolonged turnaround times (2–6 weeks). In light of these numerous shortcomings of traditional bacteriological detection methods, the WHO revised the diagnostic criteria for tuberculosis in 2025 (36), incorporating patients with positive results of molecular biological detection into the category of etiologically confirmed cases. This study aims to provide different testing protocols for rapid TB detection in various scenarios by comprehensively evaluating the diagnostic performance and health economics of existing TB detection platforms in our laboratory.

Through cross-priming amplification, EasyNAT offers rapid (<2 h), low-cost testing with room-temperature-stable reagents (8). However, in actual testing, compared with other methods, EasyNAT is currently more prone to requiring retests because of abnormalities in internal control detection. On the basis of the AUCs and DeLong test results, EasyNAT and RT-qPCR demonstrate diagnostic efficacy that is on par with that of mNGS, Xpert, and ddPCR with negative predictive values of 66.7% and 63.9%, respectively, which are higher than that of Xpert (57.1%). EasyNAT indicates potential in ruling out diagnoses.

Xpert MTB/RIF enables simultaneous MTBC detection and rifampicin resistance screening within 2 h, with enhanced sensitivity in smear-negative sputum (78.9% vs 66.1%) (15). While it has the highest specificity and positive predictive value, its utility is constrained by high costs, limited extrapulmonary TB detection, and infrastructural requirements. Therefore, it is most suitable for regions with a high incidence and burden of tuberculosis, as well as for laboratories with intermediate and above capacity to operate.

The study validated ddPCR’s efficacy in targeting MTBC conserved sequences (IS6110/IS1081), achieving a detection limit as low as that of single-copy DNA. Compared with RT-qPCR, ddPCR exhibited superior consistency (*κ* = 0.82) in low-concentration samples from smear-negative pulmonary tuberculosis and extrapulmonary tuberculosis cases. Furthermore, studies have indicated that ddPCR achieved sensitivities of 90.5% in pleural fluid and 98.5% in paraffin-embedded tissues (27, 37). Additionally, ddPCR is resistant to inhibitors, which paves the way for monitoring subclinical infections and conducting therapeutic evaluations by dynamically tracking changes in bacterial DNA load to assess treatment efficacy (3). The key advantages of ddPCR include high sensitivity, absolute quantification capability, strong resistance to interference, and applicability to diverse sample types (such as blood and lymphoid tissue) (25, 38). However, its drawbacks are also notable, including high costs for equipment and reagents, complex and time-consuming operation, low detection throughput, and a strong reliance on skilled technicians (39). In this study, ddPCR exhibited high performance advantages in various aspects. Overall, the current ddPCR method has numerous applications in both oncology and infectious diseases, and it is still under continuous development. However, owing to its relatively high cost and long turnaround time, it has not been widely adopted in the field of tuberculosis infection. Owing to its excellent comprehensive diagnostic performance, ddPCR is expected to be more widely applied in regions or laboratories with abundant medical resources in the future.

In this study, mNGS demonstrated extremely high sensitivity (100%) and negative predictive value (100%). Although it may be affected by factors such as sample size, sample selection bias, and false positives leading to falsely high sensitivity, mNGS still has the highest diagnostic performance compared with other methodologies, with an AUC of 0.971. The unbiased detection capability of mNGS, which allows for the simultaneous identification of MTBC, non-tuberculous mycobacteria, and various pathogens in mixed infections (such as viral, fungal, and other bacterial pathogens), critically impacts therapeutic strategies in immunocompromised populations (19). Laboratories can provide mNGS results within 24‒72 h, thereby expediting clinical decision-making (22) and contributing to the formulation of more precise and targeted treatment approaches. Additionally, mNGS has significant diagnostic advantages for diverse sample types, including tuberculous pleurisy, tuberculous meningitis, and spinal tuberculosis (40). However, its limitations include high costs, which restrict its application in economically underdeveloped regions (13), and the risk of false positives arising from environmental or operational contamination (41, 42), which can lead to misdiagnoses and its relatively lower specificity (75.6%) and susceptibility to false positives in cases with low reads and bioinformatics dependency (20, 23). In this study, all mNGS findings must be interpreted strictly within the clinical context (presenting symptoms, epidemiology, host immune status, and imaging findings). False-positive cases can be verified through targeted confirmatory tests (e.g., specific PCR assays for the *Mycobacterium tuberculosis* complex, ddPCR, or IGRA), effectively preventing the initiation of unnecessary antimicrobial therapy or inappropriate invasive diagnostic procedures based solely on mNGS results (29).

Now that we are aware of the pros and cons of these seven methods, how feasible is it for impoverished regions to possess all seven? Poverty (characterized by low GDP) is significantly associated with higher TB incidence, indirectly highlighting challenges in detection, prevention, and healthcare accessibility in economically disadvantaged areas. The distinct trends in Western and Central China further suggest that poverty and inequitable resource allocation may exacerbate TB transmission and underdiagnosis risks (7). Targeted interventions are needed to address gaps in healthcare infrastructure and equitable resource distribution.

The WHO’s End TB Strategy emphasizes equitable access to rapid diagnostics as a cornerstone for global elimination efforts. However, stark disparities persist: impoverished regions often lack infrastructure for molecular assays such as Xpert MTB/RIF or mNGS, whereas overreliance on AFS perpetuates underdiagnosis (7). Our study highlights actionable solutions through tiered diagnostic algorithms. In undeveloped regions with limited resources, the combination of AFS + EasyNAT + chest X-ray offers a pragmatic approach. EasyNAT’s low cost (1/35th of mNGS), room-temperature-stable reagents, and minimal technical requirements make it ideal for point-of-care testing, mitigating false positives from non-tuberculous mycobacterial infections and *Nocardia*. In contrast to RT-qPCR, which requires a specialized PCR laboratory for testing, EasyNAT serves as a point-of-care testing device with relatively portable instrumentation, reagents that can be stored at room temperature, a short testing duration, and a low risk of post-testing contamination. Moreover, the testing cost of EasyNAT is only 1/35th that of mNGS.

For primary healthcare settings (e.g., community and township hospitals), the combination of IGRAs + RT-qPCR can leverage China’s growing primary laboratory infrastructure and declining IGRA costs; IGRAs, when combined with AFS and conventional molecular testing such as RT-qPCR or EasyNAT, can be utilized for screening among the general population at the primary healthcare level (32).

The integration of Xpert MTB/RIF into routine workflows offers rapid solutions for drug resistance screening for intermediate-resource laboratories (e.g., county-level hospitals and secondary hospitals). On the basis of these fundamental tests, tertiary hospitals or specialized centers can adopt mNGS for patients with complex mixed infections, and ddPCR can be leveraged for therapeutic monitoring. In addition, ddPCR is helpful in confirming low-sequence mNGS results to mitigate overdiagnosis.

While this study advances TB diagnostics, limitations exist, such as the constraints in sample size and the absence of some detection data. Additionally, incorporating research on tuberculosis drug resistance would be beneficial. Targeted next-generation sequencing (tNGS) exhibits high sensitivity and specificity, offers lower costs, enables faster detection, and is not affected by host nucleic acid interference (43, 44). It is also currently widely used in the field of tuberculosis diagnosis (45), yet it was not included in this study. Given that our laboratory initiated tNGS testing relatively late, the remaining sample volume is insufficient to conduct supplementary tests. We will incorporate relevant comparative analyses in future research. In the future, endeavors should focus on artificial intelligence-driven bioinformatics pipelines to increase the specificity of mNGS, reduce the turnaround time, and perform multicenter validation to optimize the cost-effectiveness ratio across diverse healthcare ecosystems.

**Fig 5:**
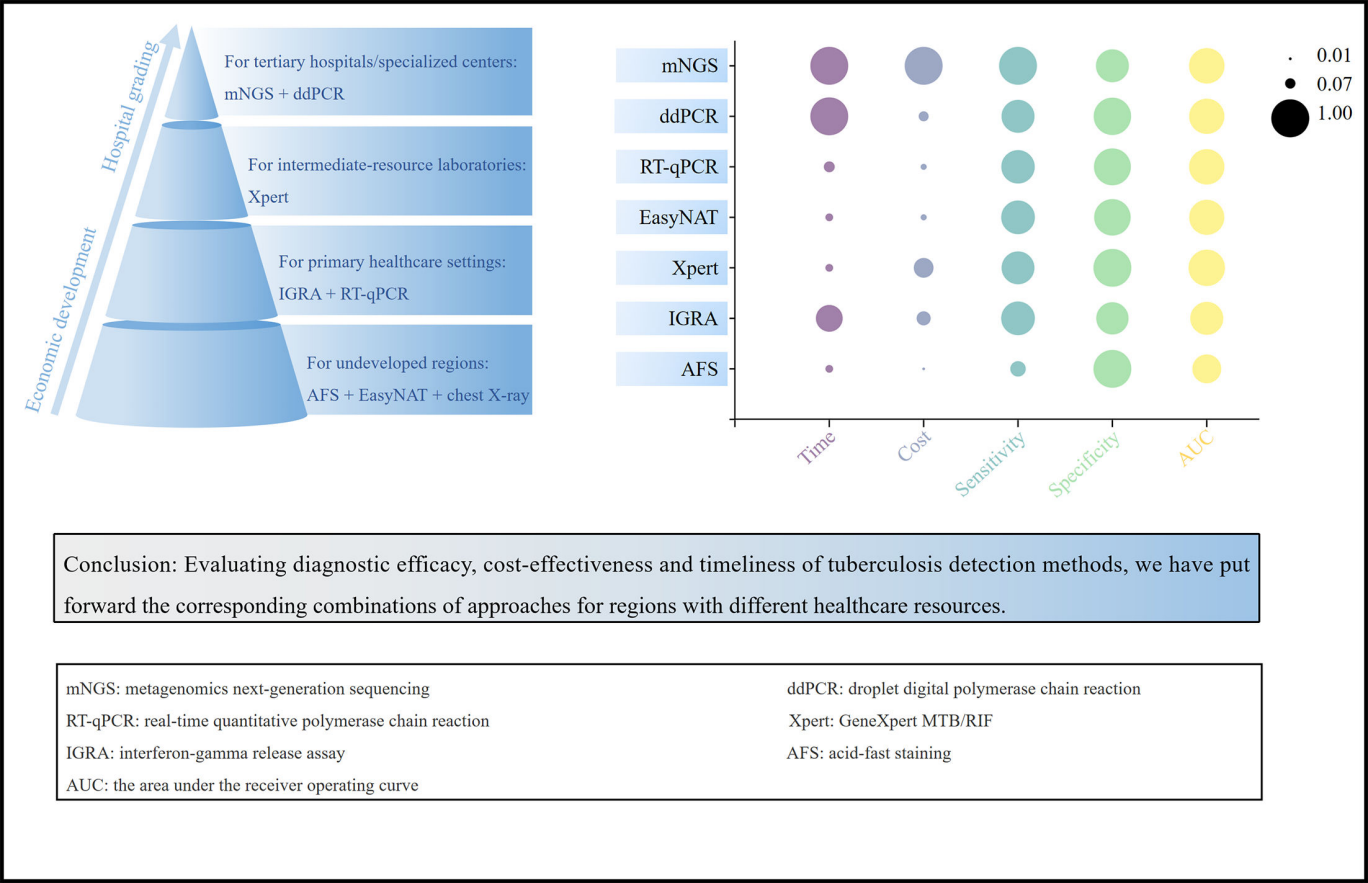
Tiered diagnostic recommendations for tuberculosis.

Through a comprehensive evaluation of the diagnostic efficacy, cost-effectiveness, and timeliness of tuberculosis detection methods, we have put forward the corresponding combinations of TB testing approaches for regions with different healthcare resources. For undeveloped regions with limited resources, a combination of AFS + EasyNAT + chest X-ray is recommended. Primary care facilities may additionally employ IGRAs + RT-qPCR. Intermediate-level hospitals can incorporate Xpert MTB/RIF for drug resistance testing, while tertiary hospitals or specialized centers should, on the basis of these fundamental tests, utilize mNGS for diagnosis and ddPCR for therapeutic monitoring in patients with complex mixed infections (Fig. 5). This strategy aims to accelerate the elimination of tuberculosis.

### Conclusion

Building upon the tailored combination testing strategies for regions with varying resources, the future of TB control hinges on their widespread implementation and continuous refinement. We expect that speeding up the development and making innovative diagnostic technologies more accessible, like more sensitive and fast molecular diagnostics and AI-aided interpretation, will further improve the accuracy and efficiency of diagnosis, especially bringing benefits to areas with limited resources. Concurrently, deepening global collaboration and knowledge sharing to ensure the large-scale application and dynamic adjustment of the most cost-effective strategies will be central drivers in bridging the final gap in early TB detection and ultimately achieving the global goal of TB elimination.

## Data Availability

The data sets used and/or analyzed during the current study are available from the corresponding author upon reasonable request.
